# Profiling of miRNAs in Mouse Peritoneal Macrophages Responding to *Echinococcus multilocularis* Infection

**DOI:** 10.3389/fcimb.2020.00132

**Published:** 2020-04-03

**Authors:** Xiaola Guo, Yadong Zheng

**Affiliations:** ^1^State Key Laboratory of Veterinary Etiological Biology, Key Laboratory of Veterinary Parasitology of Gansu Province, Lanzhou Veterinary Research Institute, Chinese Academy of Agricultural Sciences, Gansu, China; ^2^Jiangsu Co-innovation Center for Prevention and Control of Important Animal Infectious Diseases and Zoonoses, Yangzhou University College of Veterinary Medicine, Yangzhou, China

**Keywords:** *Echinococcus multilocularis*, peritoneal macrophages, microRNA, mmu-miR-155-5p, IKBKE

## Abstract

Alveolar echinococcosis (AE) is a zoonotic helminthic disease caused by infection with the larval of *Echinococcus multilocularis* in human and animals. Here, we compared miRNA profiles of the peritoneal macrophages of *E. multilocularis*-infected and un-infected female BALB/c mice using high-throughput sequencing. A total of 87 known miRNAs were differentially expressed (fold change ≥ 2, *p* < 0.05) in peritoneal macrophages in mice 30- and 90-day post infection compared with ones in un-infected mice. An increase of mmu-miR-155-5p expression was observed in peritoneal macrophages in *E. multilocularis*-infected mice. Compared with the control group, the production of nitric oxide (NO) was increased in peritoneal macrophages transfected with mmu-miR-155-5p mimics at 12 h after transfection (*p* < 0.001). Two key genes (CD14 and NF-κB) in the LPS/TLR4 signaling pathway were also markedly altered in mmu-miR-155-5p mimics transfected cells (*p* < 0.05). Moreover, mmu-miR-155-5p mimics suppressed *IL6* mRNA expression and promoted *IL12a* and *IL12b* mRNA expression. Luciferase assays showed that mmu-miR-155-5p was able to bind to the 3′ UTR of the *IKBKE* gene and decreased luciferase activity. Finally, we found the expression of *IKBKE* was significantly downregulated in both macrophages transfected with mmu-miR-155-5p and macrophages isolated from *E. multilocularis*-infected mice. These results demonstrate an immunoregulatory effect of mmu-miR-155 on macrophages, suggesting a role in regulation of host immune responses against *E. multilocularis* infection.

## Introduction

Alveolar echinococcosis (AE) is a serious life-threatening disease caused by *Echinococcus multilocularis* metacestodes in humans. AE is more prevalent in the northern hemisphere, including parts of central Asia and China (Weiss et al., 2010). In China, this disease is particularly high in pasturing regions of Northwest China, such as Xinjiang, Qinghai, and Gansu (Craig, 2006). *Echinococcus multilocularis* completes its entire life cycle in two different hosts: an intermediate host (rodents) and a definitive host (foxes or wild canids) (Kamiya and Sato, 1990). The adult *E. multilocul*aris inhabits in the small intestine of domestic and wild carnivorous canids and disperses a large number of eggs into environment with host's feces (Mackenstedt et al., 2015). Grazing farm animals or humans were infected by peroral ingestion of the food or water contaminated with *E. multilocularis* eggs (Federer et al., 2015). AE is usually diagnosed and treated in a late stage and surgery is only effective treatment (Lointier et al., 1990). Up to now, our understandings of the interactions between *E. multilocularis* and its hosts are limited, and researches in this respect are urgently needed. As key post-transcriptional regulators, miRNAs play a regulatory roles in various physiologic and pathologic processes, including host-pathogen interactions (O'Connell et al., 2010; Britton, 2017). Using high-throughput sequencing and RNA microarray, host (tissue or circulating) miRNA profiles in response to helminth infection have been widely characterized in recent years (Han et al., 2013; Hansen et al., 2016; Hong et al., 2017). For example, *Cryptosporidium parvum* infection induces the expression of *mir-30b, mir-125b-1, mir-21*, and *mir-23b-27b-24-1* in epithelial cells (Zhou et al., 2009). It was also indicated that miR-223 was significantly up-regulated in livers and sera of *schistosome*-infected mice (He et al., 2013). Our previous study found 46 miRNAs were differentially expressed in the livers of mice infected with *E. multilocularis* (Jin et al., 2017). In another study, we found circulating miRNAs in sera from *E. multilocularis*-infected mice were significantly dysregulated (Guo and Zheng, 2017a). Our recent study also showed that miRNA expression was disturbed in *E. multilocularis*- treated RAW264.7 macrophages (Guo and Zheng, 2017b). Moreover, some of altered miRNAs (miR-146a-5p and miR-155-5p) were regulators of immune and inflammatory response genes (Guo and Zheng, 2017b).

Macrophages are important for modulating immune system in host defense helminth infection (Reyes and Terrazas, 2010). However, the dynamic miRNA profiles in peritoneal macrophages against *E. multilocularis* infection remain unclear. Considering the important role of miRNAs in immune responses, we characterized miRNA profiles of peritoneal macrophages of *E. multilocularis*-infected mice and found some of altered miRNAs were related to the regulation of immune responses. These findings will provide rich resources for further studies on the functions of host miRNAs against *E. multilocularis*, which will help us to better understand the host defense mechanisms.

## Materials and Methods

### Ethics Approval and Consent to Participate

Animal experiments in the study were performed at Lanzhou Veterinary Research Institute, Chinese Academy of Agricultural Sciences and handled in accordance with good animal practice according to the Animal Ethics Procedures.

### Parasite Infection

*Echinococcus multilocularis* protoscoleces were obtained from the hydatid cysts of infected mouse in our lab. Sixty female BALB/c mice (4–6 weeks old) were purchased from Experimental Animal Center, Lanzhou Veterinary Research Institute, and were randomly divided into two groups. One group (*n* = 30) was intraperitoneally injected with 1,000 *E. multilocularis* protoscoleces as previously described (Guo and Zheng, 2017a). The other (*n* = 30) was inoculated with 0.85% NaCl solution as an uninfected group.

### Peritoneal Macrophages Isolation, Culture, and Transfection

Peritoneal macrophages were recovered from female BALB/c mice sacrificed under sterile conditions at 30-, 60-, and 90-day post infection, respectively. Cells were centrifuged at 500 g for 15 min. After removing the supernatant, the cell pellets were resuspended in RPMI-1640 medium (11875093-Gibco) supplemented with 10 % fetal bovine serum (10099141-Gibco). Isolated macrophages were seeded into 6-well plates (costar, corning incorporated) with 5 × 10^6^ cells per well and cultured in a 5% CO_2_ humified incubator at 37°C. Mouse peritoneal macrophages were, respectively, transfected with mmu-miR-155-5p mimics and miRNA mimic negative-control (NC) (Ambion/Invitrogen) using Lipofectamine™ RNAiMAX transfection Reagent (Invitrogen^TM^) according to the manufacturer's protocol (Yahiro et al., 2012). The transfection medium was replaced with RPMI-1640 medium supplemented with 10 % FBS 10 h post-transfection.

### Deep Sequencing and Data Analysis

Peritoneal macrophages from three mice in each group were randomly selected. Total RNA was isolated from mouse peritoneal macrophages using TRIzol reagent (Invitrogen) according to the guidelines (Hummon et al., 2007). High-throughput sequencing of small RNA was conducted on an illumina Hiseq 2500/2000 platform in Beijing Novogene (China) (Malone et al., 2012). Raw data were firstly processed through in-hours perl and python scripts. In this step, the clean reads were obtained by removing ploy-N, 5′ and 3′ adaptor sequences, and low-quality reads. The Small RNA tags perfectly mapped to the mouse genome (http://www.ncbi.nlm.nih.gov/genome/genomes/52) were used to identify known miRNA. Mouse known miRNAs were identified by BLAST searching against the MirGeneDB database (http://www.mirgenedb.org). Relative expression levels of miRNAs in three groups were analyzed using DESeq R package (1.8.3) (Likun et al., 2010) and differential expressed miRNAs were identified by using a fold change cut-off of ≥2 and significance *p* < 0.05. Volcano plots were used to visualize distinct expression profiles of miRNAs between two groups.

### Quantitative RT-PCR Analysis

The miRNA expression levels were examined using All-in-One^TM^ miRNA detection mix (GeneCopoia) according to the manufacturer's protocol as previously described (Guo and Zheng, 2017b). The miRNA expression levels were quantified based on the threshold cycle (Ct) values. U6 snRNA was selected as an endogenous control.

For examining mRNA expression levels, the first-strand cDNA was synthesized using 1 μg of total RNA by a ThermoScript™ RT-PCR System (Invitrogen). The reaction mixtures (20 μL) were incubated at 42°C for 1 h and then stopped by heating 75°C for 5 min. The cDNA mixture was diluted by 7-fold with nuclease-free water. Quantitative RT-PCR was performed with SYBR® Premix Ex TagTM II (TaKaRa) using 7500 Real Time PCR System (Applied Biosystems) the under following conditions: 95°C for 10 min, followed by 40 cycles of 95°C for 10 s, 60°C for 1 min. *Gapdh* was selected as an endogenous reference gene. The qRT-PCR primers were purchased from GeneCopoeia. The relative expression levels of miRNA or mRNA were calculated using the 2^−Δ*ΔCt*^ formula (Manzanoromán and Sileslucas, 2012). Statistical analysis data were taken from three independent experiments.

### Plasmid Construction and Luciferase Assay

For luciferase plasmid construction, the 3′ UTR fragment of *IKBKE* with restriction enzyme sites was amplified by RT-PCR using a pair of primers: 5′-GAGCTCGCCATTGGCCATTGGCC-3′ (restriction enzyme sites were underlined) and 5′-CTCGAGCAGCCAGTTTAGGTAATAAAC-3′ with the following steps: 94°C for 5 min, 35 cycles of 94°C for 30 s, 54°C for 30 s and 72°C for 1 min, and then 72°C for 10 min. The PCR product was subcloned into PmirGLO Dual-Luciferase vector (Promega, USA) and sequenced. The full length of *IKBKE* 3′UTR with mutations in a binding site was artificially synthesized (BGI Genomics, China), and was also subcloned into PmirGLO Dual-Luciferase vector and sequenced.

HEK293T cells were plated into 24-well plates and transfected with 1 μg WT-*IKBKE* or Mut-*IKBKE* in combination with 30 pmol mmu-miR-155-5p mimic or negative control (NC) mimic (ThermoFisher Scientific) using Lipofectamine 2000 (ThermoFisher Scientific). The luciferase activity was measured 24 h post transfection using a Dual-Glo Luciferase Assay System (Promega). Fluorescent intensity was recorded using GloMax96 (Promega). Each transfection was independently repeated three times.

### Determination of NO Secretion

Mouse peritoneal macrophages were transfected with mmu-miR-155-5p mimics or NC mimic for 12 h, followed by stimulation with 10 ng/mL IFN-γ and 100 ng/mL LPS for 12 h. The culture supernatants were harvested and nitrite levels were measured using Griess reagent (Invitrogen) (Zheng et al., 2016). Each supernatant sample was detected in triplicate and the data for final statistical analysis were taken from three independent experiments.

### Statistical Analysis

All statistical analyses were performed using GraphPad Prism 5 (La Jolla, USA), and a one-tailed unpaired *t*-test or one-way ANOVA was used for comparing differences among two or more independent groups. A *p* < 0.05 was statistically significant.

## Results

### Identification of Differentially Expressed miRNAs in Mouse Peritoneal Macrophages Against *E. multilocularis* Infection

**Figure 1:**
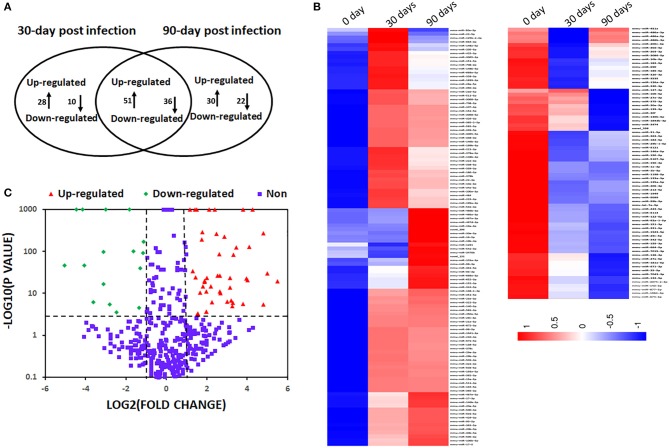
Differentially expressed miRNAs in mouse peritoneal macrophages during *Echinococcus multilocularis* infection. **(A)** Commonly shared and specific miRNAs between two *E. multilocularis*-infected libraries. Compared with the normal group, 125 and 139 miRNAs were differentially expressed in peritoneal macrophages in mice 30 and 90-day post infection of *E. multilocularis*, respectively. A total of 87 differentially expressed miRNAs were commonly shared between peritoneal macrophages in mice 30 and 90-day post infection. **(B)** A heat map of differentially expressed miRNAs between peritoneal macrophages in mice 30 and 90-day post infection. **(C)** A volcano plot of the differentially expressed miRNAs between peritoneal macrophages in mice 30 and 90-day post infection, where the red and green indicate significantly upregulated miRNAs and downregulated miRNAs, respectively.

To understand the dynamic of miRNA expression at early (30 days) and late (90 days) time points post infection, we compared global miRNA abundance of peritoneal macrophages in *E. multilocularis*-infected and un-infected mice. A total of 995 known miRNAs were identified from three libraries. Compared with the uninfected group, 125 and 139 known mature miRNAs were differentially expressed in peritoneal macrophages in mice 30- and 90-day post infection of *E. multilocularis* (Tables S1, S2), respectively. Of them, 87 differentially expressed miRNAs were commonly shared in peritoneal macrophages in mice 30- and 90-day post infection (*p* < 0.05 and fold change ≥ 2 or≤ -2), with 51 miRNAs being upregulated and 36 downregulated (Figure 1).

The predicted target genes of 87 differentially expressed miRNAs were classified according to GO functional annotations (Table 1 and Figure S1). Go analysis revealed that they were highly enriched in metabolic process, regulation of signaling, protein binding, and intracellular organelle (Figure S1). The KEGG pathway analysis indicated that top 20 pathways were highly represented, including MAPK signaling pathway, pathways in cancer, regulating pluripotency of stem cells, and Rap1 signaling pathway (Figure S2).

**Table 1:** Summary of the top 20 differentially expressed miRNAs with high abundance and small *p* value.

**miRNA**	**log2 (Fold change)**		***p*** **value**		**Predicted function^**a**^**
	**30-day post infection**	**90-day post infection**	**30-day post infection**	**90-day post infection**	
**UP-REGULATED**					
mmu-miR-672-5p	4.26	3.76	0	1.56E-252	Cell adhesion molecules
mmu-miR-199a-3p	3.79	2.45	0	5.77E-280	TGF-beta signaling pathway
mmu-miR-155-5p	2.4	1.32	0	0	T/B cell receptor signaling pathway
mmu-miR-22-3p	2.13	1.4	0	1.23E-249	Endocytosis
mmu-miR-21a-5p	1.51	1.35	0	0	Cytokine-cytokine receptor interaction
mmu-miR-23a-3p	1.41	1.37	0	0	Ras signaling pathway
mmu-miR-146b-5p	1.16	1.6	0	0	TGF-beta signaling pathway
mmu-miR-223-3p	1.79	1.79	3.18E-293	4.08E-112	Proteoglycans in cancer
mmu-miR-365-3p	4.8	4.52	2.83E-271	1.56E-252	Valine, leucine and isoleucine degradation
mmu-miR-339-5p	2.49	1.98	1.04E-266	1.72E-179	Fatty acid elongation
mmu-miR-194-5p	1.75	1.55	5.29E-193	1.36E-187	Arachidonic acid metabolism
**DOWN-REGULATED**					
mmu-miR-146a-5p	−4.46	−3.79	0	0	T/B cell receptor signaling pathway
mmu-miR-10b-5p	−4.16	−2.72	0	0	ErbB signaling pathway
mmu-miR-3535	−3.00	−1.60	0	0	T cell receptor signaling pathway
mmu-miR-30d-5p	−1.60	−2.17	0	0	T cell receptor signaling pathway
mmu-miR-150-5p	−1.24	−1.18	0	0	Central carbon metabolism in cancer
mmu-miR-185-5p	−1.76	−1.76	8.10E-304	9.06E-125	Metabolic pathways
mmu-miR-423-5p	−1.29	−1.70	1.18E-294	0	Insulin secretion
mmu-miR-151-3p	−1.14	−1.70	3.39E-172	1.97E-280	Metabolic pathways
mmu-miR-1198-5p	−1.60	−1.84	1.07E-141	7.61E-149	Vasopressin-regulated water reabsorption

a *The primary function of potential miRNAs targets was predicted by DIANA miRPath v3.0*.

### Dynamic Relative Expression of Differentially Expressed miRNAs in Peritoneal Macrophages of *E. multilocularis*-Infected Mice

**Figure 2:**
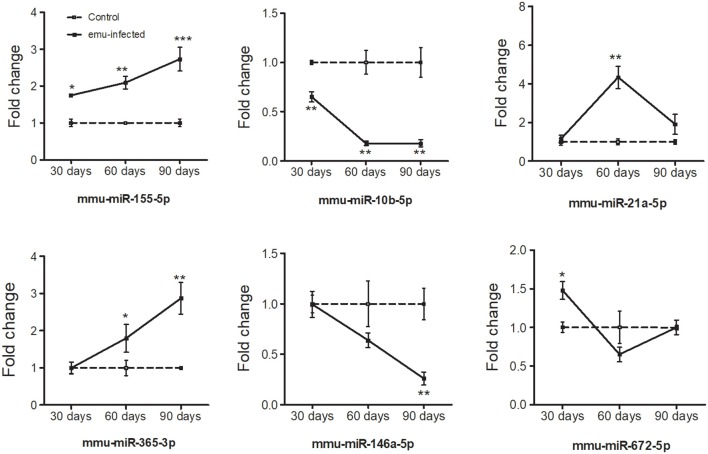
Dynamic expression of six differentially expressed miRNAs in the peritoneal macrophage cells in *E. multilocularis*-infected mice 30-, 60-, and 90-day post-infection. The expression of these miRNAs was detected by qRT-PCR and normalized to U6 snRNA. Data for final statistical analysis were taken from 3 independent experiments. *p < 0.05, **p < 0.01, ***p < 0.001.

To verify the high-throughput sequencing data, the expression levels of six selected miRNAs with high fold change and small *p* value (miR-10b-5p, miR-672-5p, miR-155-5p, miR-365-3p, miR-21-5p, and miR-146a-5p) were analyzed in peritoneal macrophages in mice 30-, 60-, and 90-day post inoculation. With the extension of *E. multilocularis* infection time, the expression levels of miR-10b-5p and miR-146a-5p exhibited a gradual reduction (Figure 2 and Table 2; *p* < 0.01), whereas the expression levels of miR-155-5p and miR-365-3p showed a gradual increase (Figure 2 and Table 2). The expression level of miR-672-5p was remarkedly upregulated 30 days post infection (Figure 2 and Table 2; *p* < 0.05) but no difference was observed between 60- and 90-day post inoculation (Figure 2 and Table 2; *p* > 0.05). The level of miR-21-5p expression was significantly upregulated 60-day post infection compared with uninfected group (Figure 2 and Table 2; *p* < 0.01).

**Table 2:** Comparison of miRNA expression obtained by qRT-PCR and RNA seq.

**miRNA**	**qRT-PCR**		**RNA seq**	
	**30-day post infection**	**90-day post infection**	**30-day post infection**	**90-day post infection**
mmu-miR-10b-5p	0.65 ± 0.06	0.18 ± 0.05	0.06	0.15
mmu-miR-672-5p	1.48 ± 0.14	1.00 ± 0.12	0.05	13.54
mmu-miR-155-5p	1.75 ± 0.06	2.73 ± 0.39	5.29	2.50
mmu-miR-365-3p	1.00 ± 0.19	2.87 ± 0.52	27.91	23.01
mmu-miR-21-5p	1.15 ± 0.25	1.91 ± 0.63	2.84	2.55
mmu-miR-146a-5p	0.99 ± 0.16	0.26 ± 0.08	0.05	0.07

### Immunomodulation of mmu-miR-155-5p on Mouse Peritoneal Macrophages

**Figure 3:**
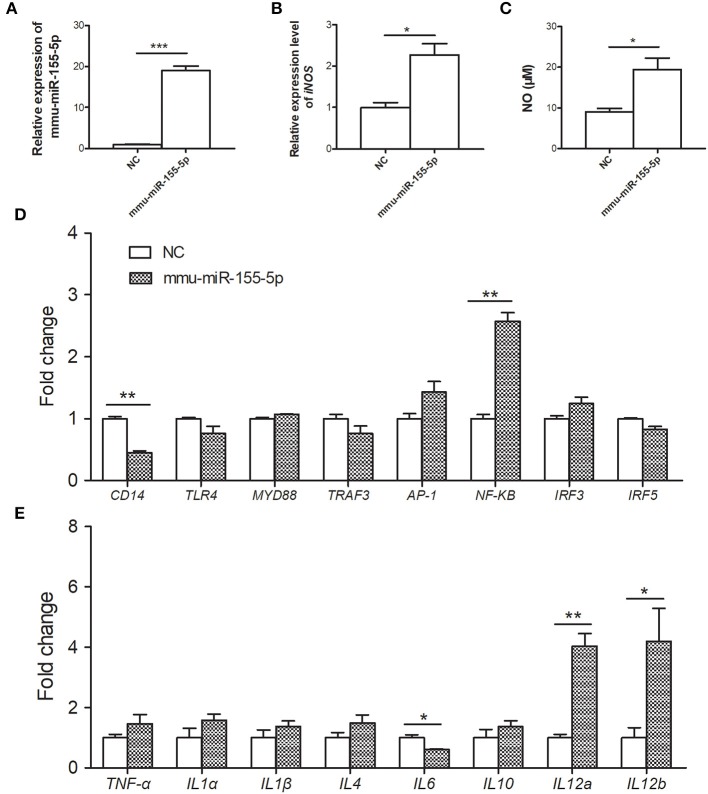
Immunoregulatory effects of mmu-miR-155-5p in peritoneal macrophage cells. **(A)** Validation of mmu-miR-155-5p expression in peritoneal macrophage cells transfected with mmu-miR-155-5p mimics. **(B)** The *iNOS* expression of peritoneal macrophages transfected with mmu-miR-155-5p mimics. **(C)** NO secretion was markedly increased in peritoneal macrophages transfected with mmu-miR-155-5p mimics. **(D)** The expression of eight genes of the TLR4 signaling pathway in mmu-miR-155-5p mimics-transfected macrophages. **(E)** The expression analysis of cytokines in macrophages over expressing mmu-miR-155-5p. Data for final statistical analysis were taken from 3 independent experiments. *p < 0.05, **p < 0.01, ***p < 0.001.

To investigate the immunoregulatory capacity of mmu-miR-155-5p on peritoneal macrophages, mmu-miR-155-5p mimics was transfected into mouse peritoneal macrophages. Compared with NC-transfected macrophages, the expression level of mmu-miR-155-5p was significantly upregulated in the mmu-miR-155-5p mimics-transfected peritoneal macrophages (*p* < 0.05, Figure 3A). In these mmu-miR-155-5p-transfected cells, it was found that the *iNOS* mRNA expression was upregulated (*p* < 0.05, Figure 3B). In agreement with the above result, compared with NC-transfected macrophages, NO secretion was increased in mmu-miR-155-5p mimic-transfected peritoneal macrophages (*p* < 0.05, Figure 3C). Moreover, the mRNA expression levels of eight genes in the TLR4 signaling pathway were also detected in mmu-miR-155-5p-transfected macrophages. Compared with NC-transfected macrophages, NF-κB was significantly upregulated, while CD14 was significantly downregulated in mmu-miR-155-5p mimics-transfected macrophages (Figure 3D). Moreover, transfection of mmu-miR-155-5p mimics increased *IL-12a* and *IL-12b* mRNA expression and decreased *IL-6* mRNA expression (Figure 3E).

### Validation of Predicted Target Genes of mmu-miR-155-5p

**Figure 4:**
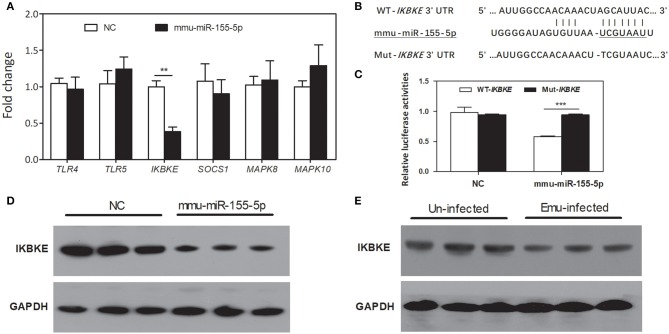
Prediction and validation of mmu-miR-155-5p target genes. **(A)** qPCR analysis of *TLR4/5, SOCS1, IKBKE*, and *MAPK8/10* mRNA expression in mmu-miR-155-5p-transfected peritoneal macrophages. Data for the final analysis were obtained from 3 independent experiments. **p < 0.01. **(B)** Schematic representation of a putative binding site for mmu-miR-155-5p in the 3′UTR of *IKBKE* gene. **(C)** mmu-miR-155-5p binding to the *IKBKE* 3′UTR *in vitro*. WT-*IKBKE* or Mut- *IKBKE* (with mutations) construct was co-transfected into 293T cells with either negative control (NC) or mmu-miR-155-5p mimics. Luciferase activities were measured at 24 h after transfection. Data for the final analysis were obtained from 3 independent experiments. ***p < 0.001. **(D)** Western blotting analysis of IKBKE protein expression in mmu-miR-155-5p-transfected peritoneal macrophages. **(E)** Western blotting analysis of *IKBKE* protein expression in macrophages from *E. multilocularis*-infected mice.

Six inflammation-related genes, including *toll-like receptor 4/5* (*TLR4/5*), *suppressor of cytokine signaling1* (*SOCS1*), *inhibitor of kappaB kinase epsilon* (*IKBKE*), and *mitogen-activated protein kinase 8/10* (*MAPK8/10*), were selected as potential target genes of mmu-miR-155-5p. Among these candidates, only *IKBKE* gene was significantly down-regulated in mmu-miR-155-5p-transfected macrophages compared with NC-transfected macrophages (Figure 4A). *IKBKE* 3′UTR contained only one putative binding site that was located at the nucleotides 146-152 (Figure 4B). Luciferase reporting assay results showed mmu-miR-155-5p was able bind to the 3′ UTR of the WT-*IKBKE* gene and significantly decreased the luciferase activity in WT-*IKBKE*-transfected HEK293T cells compared to the NC-transfected cells (Figure 4C). The repression was drastically abolished in HEK293T cells co-transfected with mmu-miR-155-5p mimics plus Mut-*IKBKE* (Figure 4C), suggesting that mmu-miR-155-5p is able to bind to the IKBKE 3′-UTR.

To further test the effect of mmu-miR-155-5p on IKBKE protein expression *in vitro*, peritoneal macrophages were transfected by mmu-miR-155-5p mimics. Western blotting results showed that mmu-miR-155-5p mimics significantly downregulated the level of IKBKE protein compared to the negative control (Figure 4D). Furthermore, we found the expression of *IKBKE* was significantly downregulated in macrophages from *E. multilocularis*-infected mice (Figure 4E). Taken together, these results indicate that mmu-miR-155-5p can repress IKBKE expression by directly targeting its 3′-UTR.

## Discussion

miRNAs are widely deemed as an important regulator of gene expression that plays a critical role in response to parasite infections (Arora et al., 2017). Increasing evidence has shown that the dysregulation of hosts miRNA expression is associated with the development of parasitic diseases, reflecting their key roles in host–pathogen interaction and immune regulation against infections (Manzanoromán and Sileslucas, 2012). miRNA profiling was mainly characterized in the liver or serum of *E. multilocularis*-infected mice (Jin et al., 2017), while the dynamics of miRNAs in peritoneal macrophages in *E. multilocularis*-infected mice remain unknown. In this study, we identified a larger member of miRNAs that were aberrantly expressed in mouse peritoneal macrophages in response to *E. multilocularis* infection. As expected, some of altered miRNAs (including miR-146a-5p, miR-155-5p, miR-21-5p, and miR-10b-5p) might be associated with immune responses (Sonkoly and Pivarcsi, 2009).

To date, increasing evidence has shown that miR-155 act as a regulator of inflammation and immune responses (Testa et al., 2017). For example, miR-155-5p is preferentially expressed in activated immune cells, such as monocytes and macrophages, and its expression is induced by type I interferons or LPS (Elton et al., 2013). Previous study showed that miR-155-5p was dramatically induced in bone marrow-derived macrophages in response to *H. pylori* infection (Elton et al., 2013). In addition, miR-155 was significantly upregulated in brains of *Toxoplasma*-infected mice during persistence infection (Cannella et al., 2014). We previously reported that the expression level of mmu-miR-155-5p was significantly upregulated in *E. multilocularis*-treated RAW264.7 macrophage cells (Guo and Zheng, 2017a). Similarly, we herein observed an increased expression of mmu-miR-155-5p in peritoneal macrophages during *E. multilocularis* infection. With the infection time prolonged, the expression of mmu-miR-155-5p was gradually increased.

miR-155-5p can regulate cytokine expression and NF-κB signaling pathway in a negative feedback loop (Yousefzadeh et al., 2015). Several studies showed that IKBKE was a major mediator of NF-κB signaling (Verhelst et al., 2013; Kim et al., 2014). In this study, we identified a potential mmu-miR-155-5p binding site in the 3′ UTR of the *IKBKE*. Moreover, we observed an inhibition of *IKBKE* expression in mmu-miR-155-5p mimics-transfected peritoneal macrophages. Interestingly, we also found that NF-κB were upregulated in mmu-miR-155-5p mimics-transfected macrophages, which is consistent with the previous finding (Zhu et al., 2016). It is well-known that NF-κB transcription factor is a major regulator of cytokine and chemokine gene transcription. We observed that upregulation of mmu-miR-155-5p could up-regulate the mRNA expression of *IL-12a* and *IL-12b* and downregulate the mRNA expression of *IL-6*. These above results suggest that mmu-miR-155-5p may modulate the expression of inflammatory cytokines possibly through the IKBKE/NF-κB signaling.

After stimulation with IFN-γ and LPS, macrophages are activated and can produce a large amount of NO (Subbanagounder et al., 2002). Excessive NO has cytotoxic effects, and it can kill intracellular bacteria, parasites and tumor cells (Oswald et al., 1994; Routes et al., 2005; Walch et al., 2015). We found that transfected mmu-miR-155-5p mimics led to an increase in NO secretion and *iNOS* mRNA expression. There is no a predictive binding site in the 3′ UTR of the *iNOS* gene, suggesting that mmu-miR-155-5p may indirectly modulate NO production. In macrophages, *iNOS* expression was induced via the NF-κB signaling (Zhang et al., 2012). In future studies, it is of interest to investigate the mechanism of mmu-miR-155-5p in NO production by macrophages.

## Conclusions

In conclusion, our study provides rich and informative data on the miRNA expression profile in peritoneal macrophages of female BALB/c mice responding to *E. multilocularis* infection. Unveiling the functions of differentially expressed miRNAs in peritoneal macrophages will provide a solid foundation for an in-depth understanding of the interactions between *E. multilocularis* and its hosts.

## Data Availability Statement

The datasets generated in this study have been deposited in the Bioproject database (accession: PRJNA609451).

## Ethics Statement

Animal experiments in the study were performed at Lanzhou Veterinary Research Institute, Chinese Academy of Agricultural Sciences and handled in accordance with good animal practice according to the Animal Ethics Procedures.

## Author Contributions

XG performed the laboratory studies. XG and YZ analyzed the data and wrote the manuscript. All authors read and approved the final manuscript.

### Conflict of Interest

The authors declare that the research was conducted in the absence of any commercial or financial relationships that could be construed as a potential conflict of interest.

## Supplementary Material

Figure S1 GO molecular function annotations of the target genes of differentially expressed miRNAs. According to *P* value, top15 GO terms of biological process, molecular function, and cellular component were shown.

Figure S2 KEGG pathway analyses of the predicted target genes of differentially expressed miRNAs.

Table S1 Summary of the differentially expressed miRNAs in peritoneal macrophages in mice 30-day post infection.

Table S2 Summary of the differentially expressed miRNAs in peritoneal macrophages in mice 90-day post infection.

## Abbreviations

NC: negative control
NO: nitric oxide
SOCS1: suppressor of cytokine signaling1
Ripk1: receptor (TNFRSF)-interacting serine-threonine kinase 1
IKBKE: inhibitor of kappaB kinase epsilon.
