# Supplementary figures and images for: Profiling of miRNAs in Mouse Peritoneal Macrophages Responding to Echinococcus multilocularis Infection
Source: Front Cell Infect Microbiol. 2020 Apr 3;10:132. doi: 10.3389/fcimb.2020.00132 (PMC7145947; doi:10.3389/fcimb.2020.00132)

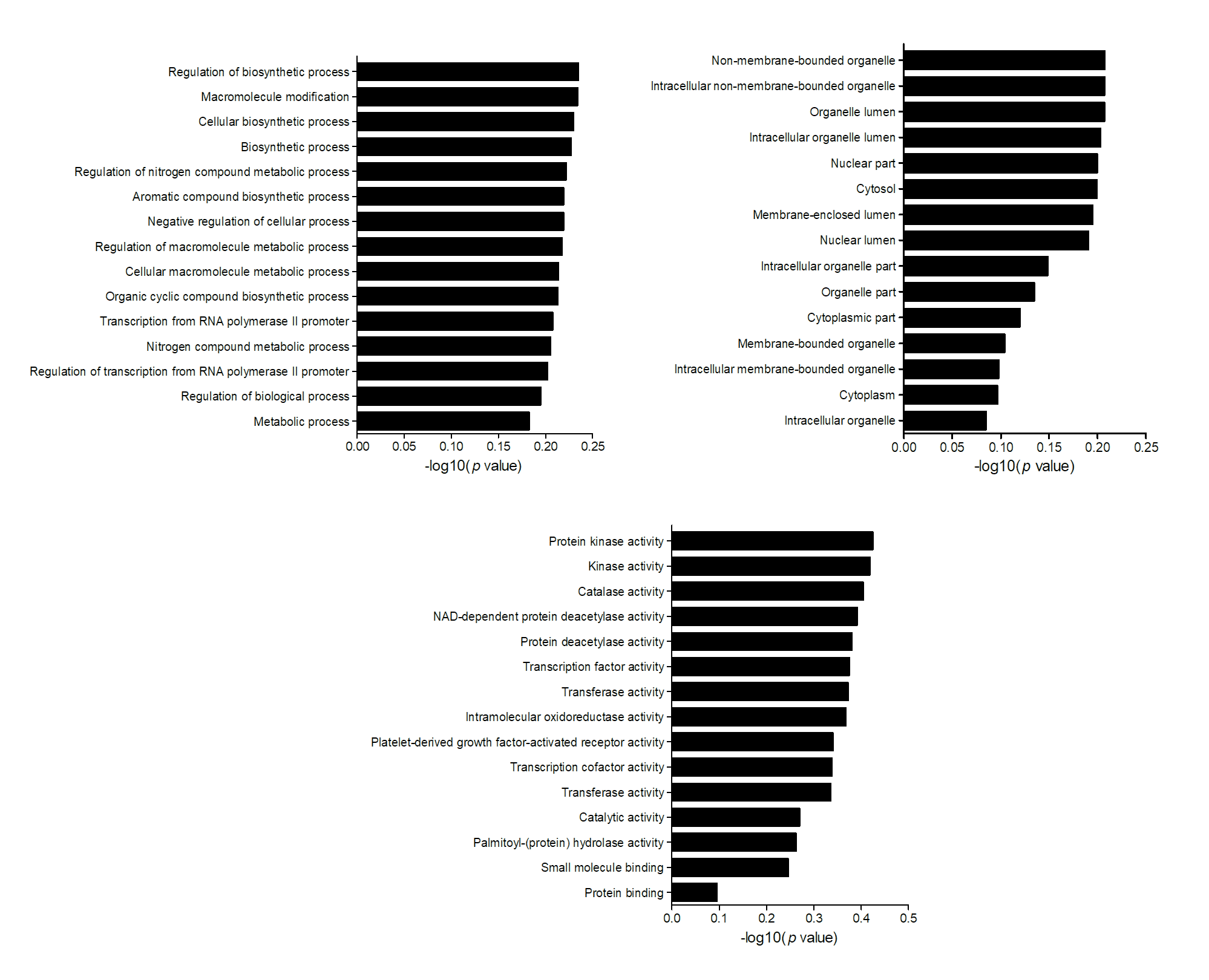

Supplement: Figure S1 — GO molecular function annotations of the target genes of differentially expressed miRNAs. According to P value, top15 GO terms of biological process, molecular function, and cellular component were shown. [file Image_1.TIF]

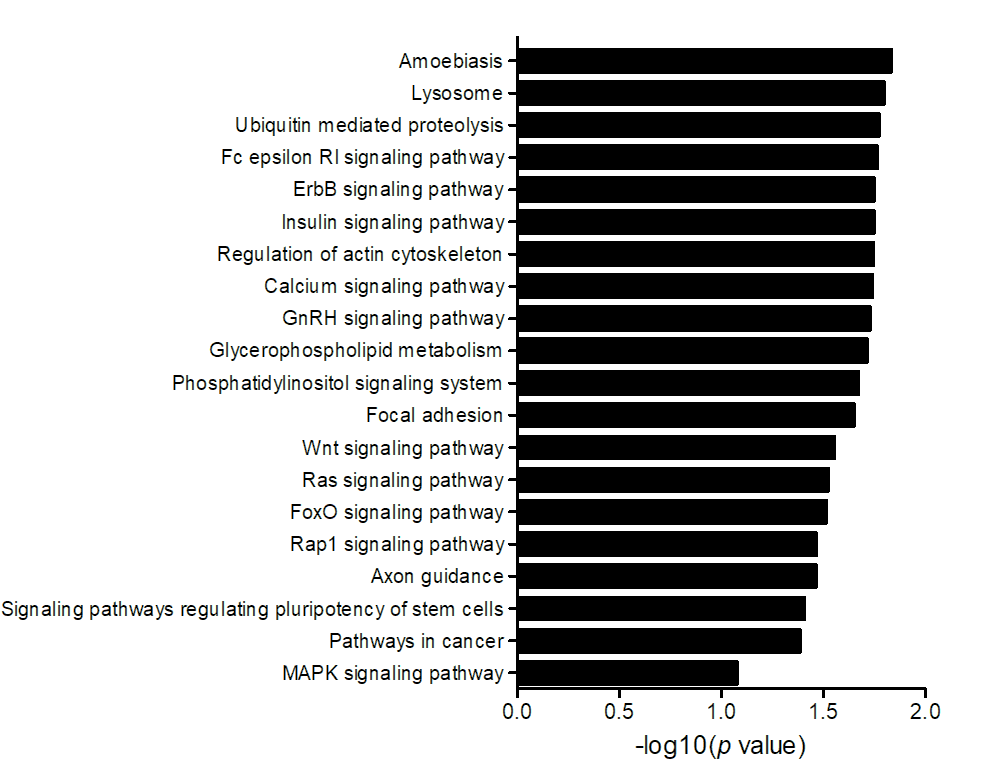

Supplement: Figure S2 — KEGG pathway analyses of the predicted target genes of differentially expressed miRNAs. [file Image_2.TIF]
