# Supplementary material for: Profiling of miRNAs in Mouse Peritoneal Macrophages Responding to Echinococcus multilocularis Infection
Source: Front Cell Infect Microbiol. 2020 Apr 3;10:132. doi: 10.3389/fcimb.2020.00132 (PMC7145947; doi:10.3389/fcimb.2020.00132)
Supplement: Table S1 — Summary of the differentially expressed miRNAs in peritoneal macrophages in mice 30-day post infection. [file Data_Sheet_1.PDF]

Table S1 Summary of the differentially expressed miRNAs in peritoneal macrophages in mice 30-day post infection

miRNA	30-day post infection	Normal control	log2(Fold_change)	*p* value
mmu-miR-10b-5p	195.49	3496.71	-4.16	0
mmu-miR-146a-5p	4559.21	100211.64	-4.46	0
mmu-miR-146b-5p	67690.64	30219.22	1.16	0
mmu-miR-150-5p	16077.41	37854.40	-1.24	0
mmu-miR-199a-3p	4309.13	312.30	3.79	0
mmu-miR-203-3p	1075.43	3785.66	-1.82	0
mmu-miR-21a-5p	120627.34	42472.13	1.51	0
mmu-miR-155-5p	4014.95	759.02	2.40	0
mmu-miR-22-3p	4879.45	1118.03	2.13	0
mmu-miR-23a-3p	9614.94	3609.26	1.41	0
mmu-miR-29a-3p	15744.36	6661.25	1.24	0
mmu-miR-30d-5p	34851.68	105612.24	-1.60	0
mmu-miR-3535	676.73	5408.80	-3.00	0
mmu-miR-672-5p	2271.09	118.51	4.26	0
mmu-miR-185-5p	985.91	3335.47	-1.76	8.10E-304
mmu-miR-423-5p	2049.11	5025.19	-1.29	1.18E-294
mmu-miR-223-3p	3312.31	955.30	1.79	3.18E-293
mmu-miR-365-3p	1192.75	42.73	4.80	2.83E-271
mmu-miR-339-5p	1975.01	351.56	2.49	1.04E-266
mmu-miR-378a-5p	1415.61	194.29	2.87	4.02E-223
mmu-miR-148b-3p	3231.23	1190.09	1.44	8.00E-208
mmu-miR-378c	4269.54	1901.90	1.17	1.37E-196
mmu-miR-30b-5p	2093.94	578.15	1.86	4.41E-195
mmu-miR-194-5p	2252.31	670.08	1.75	5.29E-193
mmu-miR-151-3p	1604.55	3542.18	-1.14	3.39E-172
mmu-miR-1198-5p	579.49	1758.54	-1.60	1.07E-141
mmu-miR-199b-5p	616.75	35.53	4.12	1.42E-131
mmu-miR-210-3p	672.65	60.87	3.47	3.23E-127
mmu-miR-150-3p	156.34	876.04	-2.49	5.25E-124
mmu-miR-222-3p	1762.93	678.03	1.38	9.07E-107
mmu-miR-320-3p	397.83	1224.62	-1.62	5.17E-101
mmu-miR-1a-3p	63.03	545.85	-3.11	1.56E-97
mmu-miR-1b-5p	63.03	545.60	-3.11	1.80E-97
mmu-miR-27a-5p	815.74	1824.88	-1.16	7.20E-92
mmu-miR-328-3p	1190.86	402.74	1.56	9.34E-88
mmu-miR-362-5p	600.16	97.89	2.62	4.95E-87
mmu-miR-138-5p	372.50	2.73	7.09	3.26E-85
mmu-miR-532-3p	461.44	45.96	3.33	5.45E-85
mmu-miR-93-5p	1788.98	869.09	1.04	2.27E-69
mmu-miR-183-5p	28.38	337.89	-3.57	3.16E-68
mmu-miR-186-5p	863.05	345.60	1.32	7.95E-50
mmu-miR-350-3p	411.80	92.18	2.16	2.65E-48
mmu-miR-200c-3p	118.34	448.21	-1.92	1.66E-47
mmu-miR-126a-3p	12.81	213.17	-4.06	2.09E-47
mmu-miR-690	5.82	194.04	-5.06	2.65E-47
mmu-miR-148a-5p	459.11	118.51	1.95	2.89E-47
mmu-miR-17-5p	606.71	214.91	1.50	8.30E-43
mmu-miR-1964-3p	262.74	651.19	-1.31	2.15E-40
mmu-miR-182-5p	58.23	294.42	-2.34	4.26E-40
mmu-miR-20a-5p	612.10	249.20	1.30	7.25E-35
mmu-miR-151-5p	507.87	195.53	1.38	5.87E-32
mmu-miR-200a-3p	541.79	219.63	1.30	2.82E-31
mmu-miR-582-3p	126.93	3.98	5.00	7.40E-31
mmu-miR-484	543.54	223.36	1.28	1.16E-30

mmu-miR-451a	305.10	637.78	-1.06	3.61E-29
mmu-miR-99b-5p	140.91	383.36	-1.44	4.87E-28
mmu-miR-146b-3p	174.68	27.83	2.65	6.34E-27
mmu-miR-500-3p	183.85	31.80	2.53	6.45E-27
mmu-miR-107-3p	361.29	132.67	1.45	6.96E-25
mmu-miR-10a-3p	216.74	53.67	2.01	4.31E-24
mmu-miR-326-3p	129.84	14.66	3.15	5.14E-24
mmu-miR-362-3p	104.22	6.71	3.96	3.71E-23
mmu-miR-98-5p	438.58	195.03	1.17	8.69E-22
mmu-miR-322-5p	93.60	5.71	4.03	3.42E-21
mmu-miR-322-3p	202.19	54.41	1.89	6.55E-21
mmu-miR-511-3p	78.17	1.74	5.49	5.88E-20
mmu-let-7e-5p	71.91	221.87	-1.63	9.69E-20
mmu-miR-29b-3p	184.14	49.19	1.90	2.56E-19
mmu-miR-22-5p	473.08	233.05	1.02	5.54E-19
mmu-miR-132-5p	21.83	121.00	-2.47	1.81E-18
mmu-miR-5121	10.19	88.45	-3.12	3.17E-17
mmu-miR-486a-3p	143.82	317.27	-1.14	6.41E-17
mmu-miR-30a-3p	421.99	208.95	1.01	6.56E-17
mmu-miR-206-3p	0.00	62.61	-6.16	1.60E-16
mmu-miR-486b-3p	141.05	308.58	-1.13	2.99E-16
mmu-miR-486a-5p	148.48	317.27	-1.10	5.86E-16
mmu-miR-155-3p	6.40	72.05	-3.49	1.36E-15
mmu-miR-15a-5p	118.34	25.84	2.20	2.56E-15
mmu-miR-3068-3p	152.99	43.98	1.80	3.45E-15
mmu-miR-339-3p	178.17	59.63	1.58	1.04E-14
mmu-miR-34a-5p	75.40	8.45	3.16	1.21E-14
mmu-miR-28a-5p	194.33	75.78	1.36	5.98E-13
mmu-miR-133a-3p	4.08	54.91	-3.75	8.06E-13
mmu-miR-140-5p	290.84	142.86	1.03	2.51E-12
mmu-miR-582-5p	44.98	0.00	6.49	5.14E-12
mmu-miR-3068-5p	101.31	25.59	1.99	7.10E-12
mmu-miR-193a-5p	70.31	11.18	2.65	9.13E-12
mmu-miR-378b	119.36	37.76	1.66	5.12E-11
mmu-miR-450a-5p	64.63	10.19	2.67	5.53E-11
mmu-miR-338-5p	135.23	48.70	1.47	1.60E-10
mmu-miR-3065-3p	128.53	44.97	1.52	1.91E-10
mmu-miR-125a-5p	62.16	149.57	-1.27	5.04E-10
mmu-miR-143-3p	88.94	185.35	-1.06	1.72E-09
mmu-miR-330-3p	12.52	60.62	-2.28	3.24E-09
mmu-miR-125b-2-3p	106.99	38.76	1.46	1.48E-08
mmu-miR-1941-3p	34.21	3.73	3.20	1.73E-07
mmu-miR-669a-5p	63.76	17.89	1.83	2.59E-07
mmu-miR-365-2-5p	34.21	4.22	3.02	3.67E-07
mmu-miR-342-5p	25.04	73.04	-1.54	4.86E-07
mmu-miR-31-5p	2.18	26.83	-3.62	7.74E-07
mmu-miR-503-5p	44.25	9.19	2.27	8.00E-07
mmu-miR-501-5p	29.11	2.98	3.29	1.06E-06
mmu-miR-1839-3p	62.74	19.88	1.66	1.96E-06
mmu-miR-511-5p	24.31	1.74	3.81	2.31E-06
mmu-miR-708-5p	20.82	0.00	4.80	3.34E-06
mmu-miR-5114	4.22	29.32	-2.80	3.99E-06
mmu-miR-425-3p	113.98	54.91	1.05	7.31E-06
mmu-miR-210-5p	24.02	2.48	3.27	9.66E-06
mmu-miR-92a-1-5p	23.58	60.13	-1.35	3.52E-05
mmu-miR-708-3p	29.99	6.71	2.16	8.10E-05
mmu-miR-664-3p	86.03	41.49	1.05	9.99E-05
mmu-miR-5099	4.66	24.60	-2.40	0.00010032

mmu-miR-205-5p	19.36	2.48	2.96	0.00015
mmu-miR-3065-5p	29.11	7.45	1.97	0.00026155
mmu-miR-338-3p	29.11	7.45	1.97	0.00026155
mmu-miR-1949	3.78	20.87	-2.46	0.00027714
mmu-miR-5107-5p	5.68	24.60	-2.12	0.00031076
mmu-miR-212-5p	4.95	22.61	-2.19	0.00040981
mmu-miR-29c-3p	36.54	12.42	1.56	0.00053139
mmu-miR-363-5p	3.64	18.88	-2.38	0.00070551
mmu-miR-29b-1-5p	7.42	25.84	-1.80	0.00087533
mmu-miR-503-3p	34.79	12.17	1.51	0.00092414
mmu-miR-148b-5p	61.43	30.06	1.03	0.0012291
mmu-miR-3086-5p	1.60	13.17	-3.04	0.0012865
mmu-miR-152-5p	34.50	13.17	1.39	0.0020179
