# Supplementary material for: Profiling of miRNAs in Mouse Peritoneal Macrophages Responding to Echinococcus multilocularis Infection
Source: Front Cell Infect Microbiol. 2020 Apr 3;10:132. doi: 10.3389/fcimb.2020.00132 (PMC7145947; doi:10.3389/fcimb.2020.00132)
Supplement: Table S2 — Summary of the differentially expressed miRNAs in peritoneal macrophages in mice 90-day post infection. [file Data_Sheet_2.PDF]

Table S2 Summary of the differentially expressed miRNAs in peritoneal macrophages in mice 90-day post infection

miRNA	90-day post infection	Normal control	log2 (Fold change)	*p* value
mmu-miR-146a-5p	7703.77	106776.23	-3.79	0
mmu-miR-10b-5p	564.11	3725.77	-2.72	0
mmu-miR-423-5p	1643.09	5354.38	-1.70	0
mmu-miR-3535	1895.70	5763.12	-1.60	0
mmu-miR-150-5p	17776.73	40334.14	-1.18	0
mmu-miR-181a-5p	4632.75	10182.74	-1.14	0
mmu-miR-155-5p	5537.97	2218.15	1.32	0
mmu-miR-21a-5p	115457.41	45254.36	1.35	0
mmu-miR-23a-3p	9930.24	3845.70	1.37	0
mmu-miR-16-5p	14597.24	5385.62	1.44	0
mmu-miR-146b-5p	97896.55	32198.80	1.60	0
mmu-miR-30b-5p	2775.94	616.02	2.17	0
mmu-miR-672-5p	1710.17	126.27	3.76	0
mmu-miR-151-3p	1106.45	3774.22	-1.77	1.97E-280
mmu-miR-199a-3p	1822.20	332.76	2.45	5.77E-280
mmu-miR-27a-5p	273.67	1944.42	-2.83	2.06E-274
mmu-miR-365-3p	1047.58	45.53	4.52	1.56E-252
mmu-miR-22-3p	3151.30	1191.27	1.40	1.23E-249
mmu-miR-194-5p	2084.81	713.97	1.55	1.36E-187
mmu-miR-128-3p	466.34	1954.48	-2.07	2.82E-184
mmu-miR-362-5p	940.54	104.30	3.17	7.14E-184
mmu-miR-339-5p	1475.03	374.59	1.98	1.72E-179
mmu-miR-1198-5p	523.08	1873.74	-1.84	7.61E-149
mmu-miR-101a-3p	1483.59	482.86	1.62	4.78E-142
mmu-miR-150-3p	125.60	933.43	-2.89	3.86E-136
mmu-miR-185-5p	1603.48	3553.97	-1.15	9.06E-125
mmu-miR-221-3p	1858.60	808.74	1.20	1.51E-120
mmu-miR-27a-3p	1631.67	3555.02	-1.12	9.28E-120
mmu-miR-93-5p	1994.90	926.02	1.11	5.20E-116
mmu-miR-223-3p	2095.16	1017.88	1.04	4.08E-112
mmu-miR-20a-5p	948.39	265.52	1.84	1.58E-106
mmu-miR-1a-3p	48.17	581.61	-3.59	2.09E-105
mmu-miR-1b-5p	48.17	581.34	-3.59	2.41E-105
mmu-miR-17-5p	870.25	228.99	1.93	1.65E-103
mmu-miR-210-3p	535.56	64.86	3.05	4.10E-102
mmu-miR-1843b-3p	108.47	659.70	-2.60	8.73E-86
mmu-miR-1964-3p	134.52	693.85	-2.37	5.39E-80
mmu-miR-199b-5p	384.99	37.86	3.35	1.47E-79
mmu-miR-19b-3p	441.01	80.74	2.45	4.15E-69
mmu-miR-152-3p	1123.22	509.60	1.14	9.74E-69
mmu-miR-138-5p	243.70	2.91	6.39	1.16E-61
mmu-miR-3074-2-3p	985.14	1971.16	-1.00	8.96E-54
mmu-miR-183-5p	53.88	360.03	-2.74	1.58E-50
mmu-miR-378a-5p	571.96	207.02	1.47	1.95E-49
mmu-miR-500-3p	259.75	33.89	2.94	5.41E-49
mmu-miR-375-3p	534.14	1230.72	-1.20	2.18E-48
mmu-miR-652-3p	614.06	242.49	1.34	2.85E-47
mmu-miR-7043-3p	261.89	760.30	-1.54	2.29E-46
mmu-miR-320-3p	621.91	1304.84	-1.07	4.73E-41
mmu-miR-582-3p	142.37	4.24	5.07	5.23E-37
mmu-miR-30e-3p	265.82	698.09	-1.39	2.92E-36
mmu-miR-532-3p	219.08	48.97	2.16	5.26E-31
mmu-miR-322-5p	120.96	6.09	4.31	3.23E-30

mmu-miR-690	30.33	206.75	-2.77	4.31E-30
mmu-miR-221-5p	360.73	799.48	-1.15	2.14E-29
mmu-miR-132-5p	7.85	128.92	-4.04	7.10E-27
mmu-miR-99b-5p	141.29	408.47	-1.53	1.57E-25
mmu-miR-126a-3p	48.88	227.14	-2.22	3.65E-25
mmu-miR-674-3p	138.80	394.18	-1.51	4.88E-24
mmu-let-7e-5p	56.38	236.40	-2.07	7.74E-24
mmu-miR-362-3p	89.56	7.15	3.65	6.08E-21
mmu-miR-143-3p	47.10	197.49	-2.07	3.56E-20
mmu-miR-182-5p	108.47	313.70	-1.53	5.45E-20
mmu-miR-511-3p	72.79	1.85	5.30	7.77E-20
mmu-miR-322-3p	182.68	57.98	1.66	1.78E-19
mmu-miR-107-3p	304.71	141.36	1.11	3.64E-19
mmu-miR-155-3p	2.50	76.77	-4.94	2.51E-18
mmu-miR-146b-3p	125.24	29.65	2.08	1.09E-17
mmu-miR-361-5p	200.17	76.77	1.38	3.96E-17
mmu-miR-326-3p	93.84	15.62	2.59	8.03E-17
mmu-miR-29b-3p	159.49	52.42	1.61	1.33E-16
mmu-miR-206-3p	1.78	66.71	-5.22	2.24E-16
mmu-miR-15a-5p	110.97	27.53	2.01	2.58E-15
mmu-miR-30f	117.39	282.20	-1.27	2.33E-13
mmu-miR-5121	16.06	94.24	-2.55	2.34E-13
mmu-miR-133a-3p	3.57	58.50	-4.04	4.87E-13
mmu-miR-106b-5p	205.52	97.95	1.07	7.47E-13
mmu-miR-130b-5p	97.05	242.23	-1.32	1.73E-12
mmu-miR-671-3p	21.77	101.92	-2.23	3.26E-12
mmu-miR-450a-5p	63.87	10.85	2.56	8.31E-12
mmu-miR-501-5p	45.67	3.18	3.85	9.38E-12
mmu-miR-10a-3p	141.29	57.18	1.31	1.02E-11
mmu-miR-139-3p	144.86	307.08	-1.08	4.22E-11
mmu-miR-193a-5p	61.73	11.91	2.37	1.01E-10
mmu-miR-342-5p	17.13	77.83	-2.18	1.92E-09
mmu-miR-669a-3p	68.51	18.53	1.89	2.16E-09
mmu-miR-3068-5p	81.35	27.27	1.58	5.20E-09
mmu-miR-3068-3p	109.18	46.86	1.22	1.08E-08
mmu-miR-125a-5p	65.65	159.37	-1.28	2.65E-08
mmu-miR-582-5p	26.40	0.00	5.72	3.84E-08
mmu-miR-100-5p	30.69	96.89	-1.66	5.46E-08
mmu-miR-25-5p	25.69	84.18	-1.71	2.00E-07
mmu-miR-5114	2.50	31.24	-3.64	3.72E-07
mmu-miR-542-3p	45.67	11.38	2.00	4.09E-07
mmu-miR-34a-5p	40.32	9.00	2.16	6.70E-07
mmu-miR-17-3p	74.22	30.71	1.27	1.23E-06
mmu-miR-92a-1-5p	17.84	64.06	-1.84	1.51E-06
mmu-miR-503-5p	39.96	9.79	2.03	1.84E-06
mmu-miR-19a-3p	29.61	4.77	2.64	2.32E-06
mmu-miR-1941-3p	27.83	3.97	2.81	2.37E-06
mmu-miR-29c-5p	30.33	82.59	-1.45	7.92E-06
mmu-miR-664-5p	44.60	105.10	-1.24	1.20E-05
mmu-miR-3470b	34.61	9.00	1.94	1.49E-05
mmu-miR-378b	80.99	40.24	1.01	1.52E-05
mmu-miR-466b-3p	36.04	10.06	1.84	1.88E-05
mmu-miR-365-2-5p	25.33	4.50	2.49	2.15E-05
mmu-miR-466a-3p	34.97	9.79	1.84	2.58E-05
mmu-miR-467e-5p	21.41	3.44	2.64	5.90E-05
mmu-miR-31-5p	5.00	28.59	-2.52	6.34E-05
mmu-miR-93-3p	26.40	6.09	2.12	7.11E-05
mmu-miR-467a-3p	37.46	12.71	1.56	8.28E-05

mmu-miR-467d-3p	37.11	12.71	1.55	9.81E-05
mmu-miR-5099	5.00	26.21	-2.39	0.00021103
mmu-miR-330-3p	26.40	64.59	-1.29	0.00035676
mmu-miR-324-5p	12.84	1.06	3.60	0.00039942
mmu-miR-1943-5p	19.27	52.15	-1.44	0.00041443
mmu-miR-1291	31.40	11.12	1.50	0.00044704
mmu-miR-29c-3p	34.61	13.24	1.39	0.00045594
mmu-miR-138-1-3p	11.77	0.00	3.89	0.00051621
mmu-miR-1949	4.28	22.24	-2.38	0.00067527
mmu-miR-511-5p	13.92	1.85	2.91	0.00069973
mmu-miR-505-3p	11.06	0.00	3.80	0.00083122
mmu-miR-210-5p	15.34	2.65	2.54	0.00085408
mmu-miR-877-5p	8.92	30.97	-1.80	0.0010556
mmu-miR-708-5p	10.70	0.00	3.75	0.0010556
mmu-miR-98-3p	9.28	0.00	5.13	0.0011636
mmu-miR-212-5p	5.71	24.09	-2.08	0.001266
mmu-miR-7015-3p	2.85	17.47	-2.61	0.0013696
mmu-miR-127-3p	13.20	37.59	-1.51	0.0017532
mmu-miR-5107-5p	7.14	26.21	-1.88	0.001823
mmu-miR-122-5p	2.85	16.68	-2.55	0.0020875
mmu-miR-503-3p	30.69	12.97	1.24	0.0021568
mmu-miR-872-3p	28.19	11.38	1.31	0.0023364
mmu-miR-3065-5p	22.84	7.94	1.52	0.0024683
mmu-miR-338-3p	22.84	7.94	1.52	0.0024683
mmu-miR-3474	0.00	9.27	-4.70	0.0026721
mmu-miR-351-5p	34.61	16.68	1.05	0.0035882
mmu-miR-20b-5p	27.12	11.38	1.25	0.0037362
mmu-miR-429-3p	27.47	11.65	1.24	0.0037772
